# Native mass spectrometry provides sufficient ion flux for XFEL single-particle imaging^1^

**DOI:** 10.1107/S1600577519002686

**Published:** 2019-04-11

**Authors:** Charlotte Uetrecht, Kristina Lorenzen, Matthäus Kitel, Johannes Heidemann, Jesse Huron Robinson Spencer, Hartmut Schlüter, Joachim Schulz

**Affiliations:** a Heinrich Pette Institute, Leibniz Institute for Experimental Virology, Martinistrasse 52, Hamburg 20251, Germany; b European XFEL GmbH, Holzkoppel 4, Schenefeld 22869, Germany; c Institute for Clinical Chemistry, University Medical Centre Hamburg-Eppendorf, Martinistrasse 52, Hamburg 20246, Germany

**Keywords:** SPI, electrospray Ionization, X-ray free-electron lasers, native MS, structural biology

## Abstract

The SPB/SFX instrument at the European XFEL provides unique conditions for single-particle imaging (SPI) experiments due to its high brilliance, nano-focus and unique pulse structure. Promising initial results provided by the international LCLS (Linac Coherent Light Source) SPI initiative highlight the potential of SPI. Current available injection methods generally have high sample consumption and do not provide any options for pulsing, selection or orientation of particles, which poses a problem for data evaluation. Aerosol-injector-based sample delivery is the current method of choice for SPI experiments, although, to a lesser extent, electrospray and electrospinning are used. Single particles scatter only a limited number of photons providing a single orientation for data evaluation, hence large datasets are required from particles in multiple orientations in order to reconstruct a structure. Here, a feasibility study demonstrates that nano-electrospray ionization, usually employed in biomolecular mass spectrometry, provides enough ion flux for SPI experiments. A novel instrument setup at the SPB/SFX instrument is proposed, which has the benefit of extremely low background while delivering mass over charge and conformation-selected ions for SPI.

## Abstract 2

The development of novel single-particle imaging injection instrumentation at the European XFEL beamline SPB/SFX is presented.

## 1. Introduction   

The first experiments at the European XFEL instrument SPB/SFX have shown new scientific possibilities especially for the life science community with successful serial femtosecond crystallography (SFX) experiments (Grünbein *et al.*, 2018 ▸). Major bottlenecks remain, especially for single-particle imaging (SPI), *i.e.* efficient sample use and interaction with the beam (Spence, 2017 ▸). The sample is generally introduced via pressure through a capillary with flow rates of several microlitres to millilitres per minute in a gas dynamic virtual nozzle (GDVN) at a concentration of 10^12^ particles ml^−1^ followed by a focusing aerodynamic lens. This approach has a relatively low sample efficiency resulting from a combination of high sample consumption and low hit rates of around 1% (Daurer *et al.*, 2017 ▸). Even though this problem is partially compensated by the high repetition rate available at the European XFEL, a lot of sample is wasted due to the pulse structure during the ‘dark time’ (see below) of the X-ray free-electron laser (XFEL). Here, we focus on new possibilities for SPI by employing nano-electrospray ionization (ESI). At a similar concentration of particles, ESI uses flow rates of 1–2 µl h^−1^ leading to a theoretically higher number of hits per sample volume. Both nano-ESI and GDVNs can be operated at lower flow rates in favourable cases; however, volumes consumed per minute in a GDVN last for an hour in nano-ESI.

The low ion densities produced by nano-ESI can be compensated by trapping the ions and pulsing their release (Myung *et al.*, 2002 ▸). This approach ideally suits the pulse structure at the European XFEL with a dark time of 99.4 ms and 0.6 ms pulse trains of up to 2700 pulses with 220 ns interspacing. The 10 Hz pulse train repetition results in up to 27 000 pulses s^−1^ with a maximum of 13 500 pulses s^−1^ at the SPB/SFX instrument. For SPI, X-ray interaction with a liquid jet is not suitable due to the water background. For fixed target holders, the background created by the support is problematic (Sun *et al.*, 2018 ▸). Moreover, for aerosol injection, high background originates from crusts of buffer components and other adducts surrounding the sample hampering high-quality data acquisition (Daurer *et al.*, 2017 ▸). Even if aerosol injectors are to be combined with ESI, the type of ion source used introduces a high gas load and ions are neutralized before entering the vacuum stages, which no longer allows the easy separation of gas and particles. Here, a nano-ESI approach without neutralization is presented that is able to remove any buffer- and gas-associated background (Tahallah *et al.*, 2001 ▸).

Even more exciting is the fact that ions created in nano-ESI can be manipulated. Commonly used techniques in mass spectrometry (MS) have the advantage of online selection and hence ‘purification’ of a specific mass, or rather mass-to-charge ratio (*m*/*z*), and even conformation before interaction with the beam. Studies suggesting a dipole orientation for molecules without rotational symmetry offer interesting options for manipulation (Thesing *et al.*, 2017 ▸). Thus, the method can compensate for sample heterogeneity and overcome the second bottleneck of data evaluation problems when samples are introduced unselected. The low number of scattered photons from single particles leads to extensive datasets and time-consuming analysis due to the necessity of sorting and assignment (Marklund *et al.*, 2017 ▸). Similar mass and conformation selection approaches have been performed for UV-action spectroscopy and photo-dissociation in MS (Bellina *et al.*, 2014 ▸; Daly *et al.*, 2018 ▸). Notably, under gentle conditions in volatile buffer surrogates, such as ammonium acetate (AmAc), nano-ESI preserves native-like conformations of non-covalent protein complexes, *i.e.* closely resembling the native solution conformations. Evidence is provided by ion-mobility MS and MS coupled to infrared spectroscopy at FELs (Chandler & Benesch, 2018 ▸; Leney & Heck, 2017 ▸; Uetrecht *et al.*, 2010 ▸; Seo *et al.*, 2016 ▸). Hence, these protein complex ions are suitable for structural studies. Similar approaches have been employed for electrokinetic injection of crystals into the XFEL beam (Sierra *et al.*, 2012 ▸).

Here, we present a feasibility study for a new proof-of-principle setup exploiting the special properties and potential of the beam for SPI. We have previously stated the potential to use native MS for sample delivery at XFELs to enable time-resolved SPI (Schulz *et al.*, 2013 ▸). In this work, the actual ion flux is determined from a conventional nano-ESI source employed in commercial time-of-flight (ToF) systems for native MS. We discuss data acquisition times for mass- and conformation-selected ions and verify that the ion flux generated is sufficient to render this approach feasible for SPI at the European XFEL. The setup used here is identical to the initial stages of the native MS instrumentation that will be used for future XFEL experiments and is also widely used in the native MS field.

## 2. Materials and methods   

**Figure 1:**
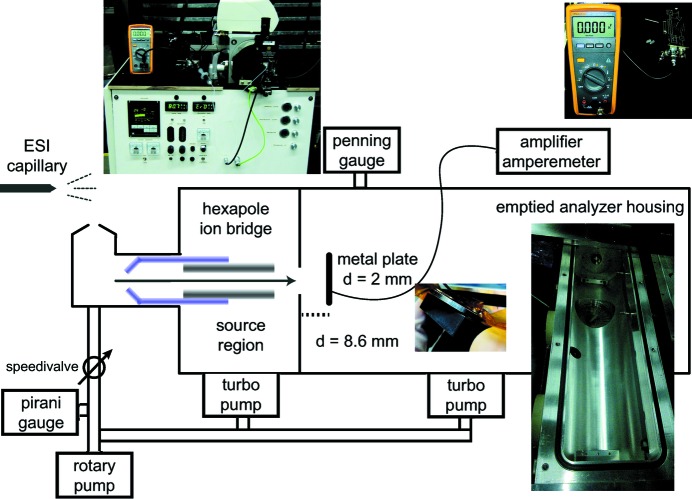
The experimental setup for ion-flux measurements.

The instrumental setup was based on the front-end of a Q-ToF (MS Vision, the Netherlands, Fig. 1 ▸). In order to assess the ion flux and density reaching the interaction point, only the nano-ESI source, pressure adjustment in the source and the first hexapole ion bridge were used by stripping the analyser housing of the quadrupole and collision cell. The ToF was replaced by a blind flange to seal the vacuum. The electronics were modified to run in standalone mode without a control computer. Ion currents were measured behind the exit orifice of the hexapole ion guide in the source region. The hexapole radiofrequency (RF) runs up to 600 V peak-to-peak amplitude. An ion-CCD (OI Analytical, US) or a 2 mm-thick metal plate connected to an amplifier [Femto DDPCA-300, Germany, with the following settings: full bandwidth, fast rise time, bias off, 10^11^ trans impedance gain (V A^−1^)] in line with an ampere meter (Fluke 233, Germany) was used to read out the ion current directly.

The capillary voltage was set to 1.5 kV and the cone voltage was set to 150 V. The vacuum pressure in the source region was set to 10 mbar in high source pressure configuration and 2.4 mbar in low-pressure configuration (valve fully open). The corresponding pressures from the penning gauge in the analyser housing were 2.3 × 10^−5^ mbar and 1.1 × 10^−5^ mbar, respectively. The hexapole RF and DC offset were ramped to identify optimal conditions. GroEL was kindly provided by Rob Meijers from the Sample Preparation and Characterization Facility, EMBL Hamburg (Boivin *et al.*, 2016 ▸). GroEL was purified using a C-terminal His-tag by affinity chromatography using Ni-NTA beads followed by size exclusion. The 14mer GroEL was sprayed at 10 µ*M* monomer in 50 m*M* ammonium acetate, pH 6.8. Separated Hepatits B virus (HBV) T3 (180mer) and T4 (240mer) capsids were provided by Norman Watts (NIH, Bethesda, USA) (Wingfield *et al.*, 1995 ▸). HBV capsids were sprayed at 8 µ*M* monomer concentration from 150 m*M* ammonium acetate, pH 6.8. Buffer exchange was performed and electrospray capillaries were produced as previously described (Garcia-Alai *et al.*, 2018 ▸; Uetrecht *et al.*, 2008 ▸; van Duijn *et al.*, 2006 ▸). All errors presented are standard deviations from three technical replicates.

## 3. Results   

### 3.1. Increased source pressure leads to improved desolvation of samples   

GroEL, a well characterized protein complex in native MS, was used for benchmark measurements and calculations (van Duijn *et al.*, 2006 ▸). GroEL is a protein complex of over 800 kDa in mass with 14 single subunits assembling in two stacked seven-subunit rings. It is often used for test purposes in native MS and, due to its size, is a suitable sample for initial SPI experiments. In order to provide a more challenging sample for MS with high suitability for SPI, in addition we used Hepatitis B virus T3 and T4 capsids, which were 3 MDa (T3) and 4 MDa (T4) in mass (Uetrecht *et al.*, 2008 ▸).

**Figure 2:**
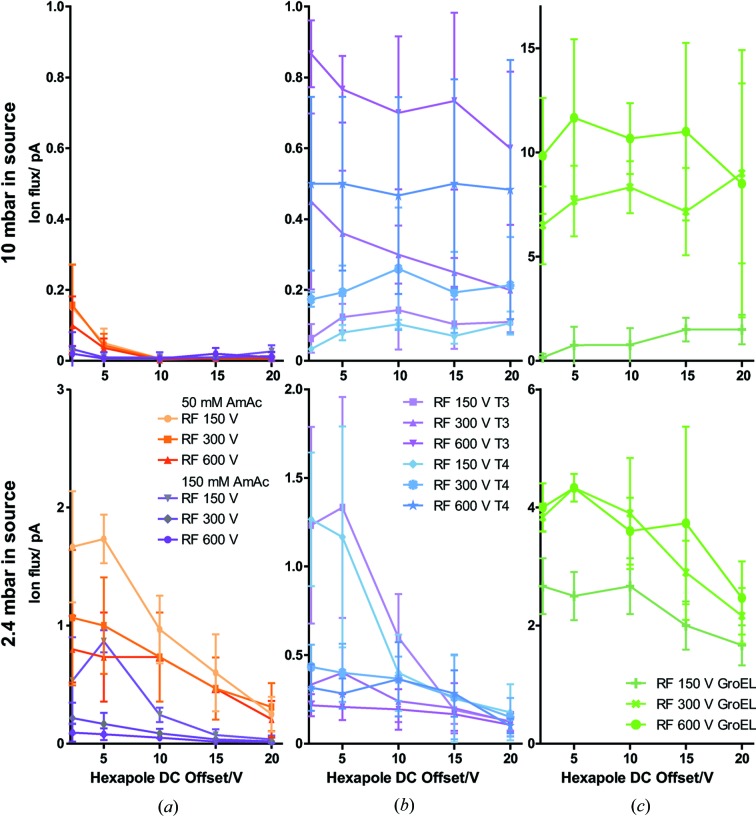
The ion flux in the source region under different conditions. The ion flux in pA with elevated source pressure (top) and no additional pressure (2 mbar, bottom) is shown for different hexapole DC offsets (*x* axis) and RF amplitudes. (*a*) Pure buffer ammonium acetate, AmAc, (*b*) T3 and T4 HBV capsids and (*c*) GroEL are compared.

The ideal conditions for the transmission of ions from the ESI source across the hexapole towards the detection point were tested using different pressure and hexapole settings in the ESI source region as found in commercial mass spectrometers. Low pressure resulted in a high transmission rate of buffer droplets or clusters as well as low transmission of free protein and capsid ions, whereas high pressure had the opposite effect (Fig. 2 ▸). This is likely due to better release of proteinaceous ions from the buffer clusters by desolvation, *i.e.* evaporation of buffer molecules, resulting in faster shrinking of droplets and droplet fission. Additionally, collisional cooling improves the transversal focusing and therefore the entry probability into the MS of the large biomolecular ions as shown previously for ion transmission towards the final ToF detector and suggested by simulations (Tahallah *et al.*, 2001 ▸; van den Heuvel *et al.*, 2006 ▸). So far, experimental proof that ion transmission in the early stages of the instrument is indeed influenced by the pressure in the source region was lacking. Without increased source pressure, no sample-specific signal was detected, suggesting that proteins are hidden in buffer clusters. Our results strongly support prior suggestions that the amount of water clusters and water surrounding particles of interest can be controlled using gas and voltage and thus reduced (Schulz *et al.*, 2013 ▸). The sample temperature can be controlled by the same means.

**Figure 3:**
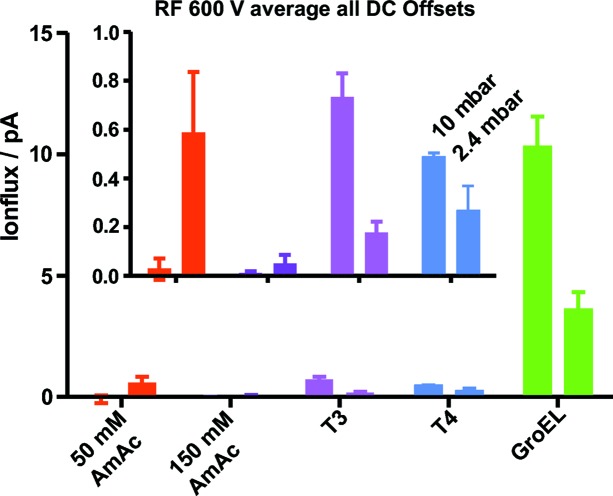
The ion flux in the source region under different source pressures at 600 V RF amplitude averaged over all DC offsets. The buffer is AmAc.

Unlike proteinaceous samples, buffer clusters are sensitive to hexapole DC offset with higher transmission at lower offsets. The highest RF amplitudes of 600 V are best for high ion fluxes. Therefore, Fig. 3 ▸ shows averages of ion flux over all DC offsets for the highest RF. The ion flux is higher for the 800 kDa protein complex GroEL than for the 3 MDa T3 and 4 MDa T4 HBV capsids with the transmission dropping by a factor of >10 for the large capsids. This decrease may be related to the overall >10 times lower particle concentration (0.71 µ*M* GroEL, 0.044 µ*M* T3, 0.033 µ*M* T4). Nevertheless, the transmission can be further improved since for GroEL, with a main charge state of 69+, a maximum current of 1.32 nA would be expected at a flow rate of 1 µl h^−1^. In turn, this also suggests that, with optimized ion transmission, sample consumption can be further reduced.

ESI in connection with modification of the source pressure (Soleilhac *et al.*, 2015 ▸) leads to a low background due to improved evaporation and ‘collisional cooling’ originating from more gas molecules in the source region. This is a prerequisite for SPI. Very few or no water clusters will make their way to the interaction region, allowing for rather easy vetoing at the diffraction pattern detector. Furthermore, a pressure of 10^−5^ mbar was used at the point of measurement, which can be further reduced at the interaction point in the final instrument setup. This shows that ESI will create low background as required for SPI and will be superior to aerosol jets in this respect.

Nano-ESI has the benefit of low sample consumption coupled with higher sample efficiency due to less sample loss; however, enough particles need to be provided in order to keep up with the repetition rate at the SPB/SFX instrument at the European XFEL and allow for decent hit rates and acquisition times. A too low ion count for the means of SPI could be problematic. We tested whether the ion count could be increased by introducing higher flow rates through applying pressure with a syringe. However, increased sample flow rate did not lead to a significant increase in the ion count. On the commercial full version of this and similar mass spectrometers, applying pressure on the back of the needle relates to worse desolvation of the sample. Poor desolvation leads to multiple adducts on each of the charge states and worse signal and resolution as shown by the readout from the ToF analyser. Mainly larger droplets were created at the tip of the capillary, thus desolvation is likely to become the limiting factor again, and the results were similar to using low pressure in the source region. This hypothesis could not be corroborated because our setup was not equipped with a mass analyser.

### 3.2. Nano-ESI coupled to MS provides sufficient ion flux for SPI experiments   

**Figure 4:**
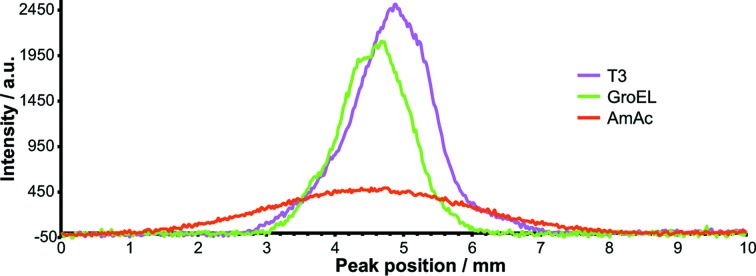
The ionCCD profile for buffer AmAc, GroEL and T3, revealing better focusing of protein complexes as opposed to buffer clusters.

Two different methods were used to monitor the ions. The ionCCD detector (OI Analytics, USA) provides information about width and shape of the ion beam (Fig. 4 ▸) that confirms theoretical calculations (van den Heuvel *et al.*, 2006 ▸). The hexapole confines the ions to a Gaussian-shaped beam with a full width at half-maximum (FWHM) of 1.2–1.32 mm for protein samples, independent of the mass (GroEL, 800 kDa: 1.2 mm; T3, 3 MDa: 1.32 mm). Less focusing is observed for buffer clusters at low source pressure (3.74 mm). Ion flux cannot be determined directly from the ionCCD detector, which requires calibration. Therefore, hit rates for SPI were calculated using the ion currents presented in Fig. 3 ▸.

The maximum achievable ion density and focusing depends on the design of the ion optics and trap, which can be further improved in future instrument design. Space–charge limits are highly influenced by applied voltages. In addition, the charge state of the ion of interest influences its behaviour and, if required, can be reduced by addition of chemicals to the buffer (Bagal *et al.*, 2009 ▸). We calculated that a minimum of 1000 ions mm^−3^ are needed in order to achieve feasible hit rates for SPI, whereas 1 000 000 ions mm^−3^ would result in multiple particle hits. The ion count behind the hexapole is approximately five times higher than that calculated from ToF signals due to improper counting of ions and ion loss. This discrepancy further implies that ion transmission efficiency at subsequent stages can still be improved.

We demonstrated an ion count corresponding to 0.93 × 10^6^ ions s^−1^ for GroEL and up to 0.033× 10^6^ ions s^−1^ for the HBV T3 capsid. Table 1 ▸ shows the ion counts detected with the plate setup in more detail. The European XFEL bunch structure is defined by a 10 Hz repetition of a 600 µs bunch train, which can contain up to 2700 pulses with a maximum of 1350 delivered to a single instrument. Pulses are between 10 fs and 100 fs in length each and spaced a minimum of 220 ns apart. In a mass spectrometer, ions can be stored on a stable trajectory and released when needed. The system we are currently designing for the purpose of SPI allows for the accumulation of ions over the ‘dark time’ of 99.4 ms that is defined by the bunch structure of the European XFEL (Table 1 ▸). Trapping over such times is straightforward but care has to be taken to maintain native-like structures (Myung *et al.*, 2002 ▸). Taking GroEL as an example, over 90 000 ions can be collected during this time in the trapping region of the mass spectrometer followed by a timed release of the stored particles.

The required acceleration and final speed of the ions depends on the focal spot of the instrument. The ion velocity can be readily changed and higher velocities correlate with better ion-focusing capabilities. SPB/SFX provides a small focus of ∼100 nm and a larger focus of ∼1 µm. Our results show that, with GroEL at high ion density and with a small focus, the entire 600 µs bunch train can be supplied with ions. There are more ions than required showing that ion losses at later stages in the setup can be compensated. In the case of HBV capsids, the current ion transmission is only sufficient to supply half or one third of the bunch train at high ion density and small focus (Table 1 ▸, condition A&D). Depending on how pulses are distributed between instruments, this may still be sufficient to supply all pulses arriving at SPB/SFX.

### 3.3. Considerations concerning hit rates   

The beam width determined with the ionCCD allows estimation of hit rates for XFEL interaction. For ease of calculation, we assumed that ions will be further focused to 1 mm (Table 2 ▸), which appears realistic from the data in Fig. 3. In the future, better focusing is possible; however, a value of 1 mm is also feasible with commercial setups. Given the mass selection capabilities, a set of less than 100 000 diffraction patterns is assumed sufficient to reconstruct a well resolved structure. Notably, low-resolution structures available so far have used only a few hundred patterns (Ekeberg *et al.*, 2015 ▸; Hosseinizadeh *et al.*, 2017 ▸; Kurta *et al.*, 2017 ▸). However, the required number of patterns is highly dependent on the sample and data quality as well as the desired resolution. Acquisition times are given for an arbitrarily chosen number of 10 000 diffraction patterns.

With a 1 µm focus, 10 000 patterns are recorded within less than half an hour maximum despite the low hit probability for each pulse, assuming that vetoing is available as the AGIPD detector cannot record all possible 1350 patterns. This short collection time highlights the advantage of high repetition rates. For the small focus of 0.1 µm diameter likely to be required for high-resolution data, data acquisition times are longer, exemplifying the need for sufficient ion density. Nevertheless, with high ion density, 10 000 patterns would be recorded in 2 h 40 min.

To test whether a similar setup could be used at LCLS II, which is currently under construction, we calculated hit rates using a repetition rate of 10 kHz. While LCLS II can in theory go up to 1 MHz, the detectors cannot record at the same speed and this limits acquisition rates to 10 kHz (Dunne, 2017 ▸). The repetition rate limits the dark time to 100 µs. Using the number of ions for GroEL would allow trapping ∼93 ions resulting in fairly low ion densities and 10 000 patterns would be acquired in less than 4 h. As outlined above, the ion flux can still be improved, which would result in more feasible hit rates. Another way to improve the acquisition times is focusing the ions to a beam width far below 1 mm. Higher flux and focusing would, in principle, even allow SPI without trapping at LCLS II using the 1 MHz repetition rate.

## 4. Discussion and outlook   

We have shown that the ion flux from nano-ESI, which is well suited for ionizing protein complexes, as used in native MS in combination with trapping to increase ion density, is indeed sufficient for SPI at the SPB/SFX instrument of European XFEL and, under certain conditions, at LCLS II. This approach will benefit from pulsing, mass and conformation selection using standard MS components such as quadrupole mass filters, digital ion traps and ion-mobility devices (Bandelow *et al.*, 2013 ▸; Uetrecht *et al.*, 2010 ▸). Nano-ESI will enable the recording of clean datasets for a small sub-ensemble and therefore smaller datasets, speeding up data analysis times.

Although in theory the setup could be used for SFX of tiny nano-crystals, the intrinsic sample heterogeneity from the crystal size would limit the benefit of ion manipulation, *e.g.* *m*/*z* selection. Moreover, a 20 × 20 × 20 molecule lysozyme crystal would already have a molecular weight of 114 MDa, posing a challenge for ion transmission. Last but not least, crystal lattice contacts are weak and it is unclear whether these would survive water removal.

The low background due to the lack of produced/transmitted buffer clusters will enable easy vetoing at the X-ray detector for diffraction pattern collection. Moreover, large protein complexes carry dipole moments, which in principle would allow orienting particles along one axis (Shvartsburg *et al.*, 2009 ▸). Recent theoretical calculations have confirmed this possibility and it would also reduce the number of patterns required for reconstruction or allow reconstruction from low-intensity or information-lacking patterns (Marklund *et al.*, 2017 ▸). Notably, HBV capsids still pose some difficulty due to limited ion flux. Nevertheless, some viral particles of even larger sizes have higher ion transmission efficiencies (Shoemaker *et al.*, 2010 ▸). Moreover, implementing new developments like the aerolens could result in greatly increased ion fluxes and therefore allow shorter acquisition times or low-intensity samples to be studied by this approach (Lekkas *et al.*, 2017 ▸). The designed proof-of-principle setup will contain a newly designed digital trap and a short ion-mobility stage to allow for maximum ion transmission in front of the interaction region. Pressures at the X-ray interaction point are foreseen to be well below 10^−5^ mbar, resulting in an overall low gas and sample load in the system. While any additional stages cause reduced ion counts, the GroEL sample shows that there are access ions that can compensate for these reductions. Intuitively, *m*/*z* selection is accompanied by losing ions of different *m*/*z*. However, the overall number of transmitted ions may still be higher than in the non-selective mode, which usually requires scanning along the entire range. Additionally, optimizing the focusing of the ion beam at the final interaction point will be a major task for instrument development to allow most ions to reside within the X-ray focus.

Implementation of such a setup is currently being developed dedicated to the SPB/SFX beamline at the European XFEL. For initial feasibility and testing purposes, a chamber in chamber setup is foreseen and includes an ESI source, a digital ion trap, ion-mobility separation and a ToF analyser.

## Figures and Tables

**Table 1:** Ion flux for protein samples at 10 mbar source pressure For each protein complex the molecular weight (*M*), main charge state (*z*) in native MS and the ion current after subtraction of the buffer signal are provided. The ion current is converted into number of ions and the amount of ions that can be trapped in the dark time (99.4 ms) is calculated. Using the beam properties at SPB/SFX, the minimal speed to refresh ions between individual pulses in a bunch train can be calculated to determine for how long ions can be refreshed. Ideally, this time should be at least 600 µs to allow use of the entire pulse train. Four different conditions are compared: (A) ion density of 1000 mm^−3^, particle speed 2 µm/220 ns for larger focus; (B) ion density of 10 000 mm^−3^, particle speed 2 µm/220 ns for larger focus; (C) ion density of 1000 mm^−3^, particle speed 0.2 µm/220 ns for smaller focus; and (D) ion density of 10 000 mm^−3^, particle speed 0.2 µm/220 ns for smaller focus [retrieves the same values as condition (A)].

Sample	*M* (MDa)	*z*	Ions (pA)	Ions (s^−1^)	Ions (dark)	A&D *t* (µs)	B *t* (µs)	C *t* (µs)
GroEL	0.8	69	10.3	931944	92635	10190	1019	101899
T3	3	135	0.7	33317	3311	364	36	3643
T4	4	158	0.5	18856	1874	206	21	2062

**Table 2:** Acquisition times for 10 000 diffraction patterns for different boundary conditions at the European XFEL and LCLS II For SPB/SFX, the hit probabilities per pulse are provided for ion densities of 1000 and 10 000 mm^−3^ also used in Table 1 ▸ using both the large and small focus XFEL beam. Using the maximum number of 13 500 pulses s^−1^, the diffraction patterns acquired per second and the time required for 10 000 are determined. The last row provides values for LCLS II. Here, a maximum repetition rate of 1 MHz can be achieved; however, the detector will only support up to 10 kHz, which has therefore been used to calculate the number of GroEL ions that would be trapped in that time and would be available for recording diffraction patterns.

Ion density (mm^−3^)	Beam (µm)	Hits per pulse	Patterns (s^−1^)	*t* for 10 000 patterns (min)
1000	1	0.00079	11	16
1000	0.1	7.9 × 10^−6^	0.11	1572
10000	1	0.0079	106	1.6
10000	0.1	7.9 × 10^−5^	1.1	157
93	1	7.3 × 10^−5^	0.73	228
